# Supplementary material for: Strengthening Community End-of-Life Care through Implementing Measurement-Based Palliative Care
Source: Int J Environ Res Public Health. 2022 Jun 24;19(13):7747. doi: 10.3390/ijerph19137747 (PMC9265763; doi:10.3390/ijerph19137747)
Supplement: Supplementary file 1 [file ijerph-19-07747-s001.zip › ijerph-1779954-supplementary.pdf]

INTERVIEW QUESTIONS

1. **What do you know about the IPOS?**

PROMPT: What is it?

Where is it used?

What is it for?

2. **Have you been using the IPOS?**

PROMPT: How regularly?

Is it ever repeated? – why/why not?

When do you use/apply it?

What method of implementation is used? (eg. read to pt, give to pt etc)

If not, why?

3. **How do you find using the IPOS?**

PROMPT: Do you find it easy/difficult to incorporate into patient appointments/conversations? - explain/whys that?

Have you had good/bad experiences utilising it? – explain/elaborate

Has it helped/hindered your practice? – in what way?

Do you find it correlates with all the health models we use in NZ? (Te Whare tapa wha, Fonofale model, Te pae mahutonga, Te wheke etc) – why/in what way?

Are you comfortable and confident asking all the questions listed? – if no, which ones and why? Do you think any further training/education is needed? – why? if yes in what way/what about?

4. **If any changes regarding the following were to be made to help your practice, what would these consist of:**

PROMPT: Layout
Structure
Any symptoms/patient circumstances that need to be addressed or added?
Any unnecessary items?
Further information/feedback

5. **Has it changed your practice at all since its introduction?**

PROMPT: If yes, In what way?

Has this been good/bad?

6. **Do you think its use benefits the patient?**

PROMPT: Why/why not?

Have you noticed any difference in the standard of care patients have received after the IPOS tool was introduced? What are these? How has it impacted Hospice as a whole?
